# The role of radiotherapy in the age of immunotherapy

**DOI:** 10.1093/jjco/hyaa268

**Published:** 2021-02-10

**Authors:** Hiro Sato, Sandra Demaria, Tatsuya Ohno

**Affiliations:** Department of Radiation Oncology, Graduate School of Medicine, Gunma University, Maebashi, Gunma, Japan; Gunma University Heavy Ion Medical Center, Maebashi, Gunma, Japan; Department of Radiation Oncology, Weill Cornell Medical College, New York, NY, USA; Department of Radiation Oncology, Graduate School of Medicine, Gunma University, Maebashi, Gunma, Japan

**Keywords:** radiotherapy, DNA damage response, immunogenic cell death, immunotherapy, immune checkpoint inhibitors

## Abstract

With the development of immune checkpoint inhibitors, the efficacy of immunotherapy as a cancer treatment that is effective against multiple tumor types has been established, and this modality came to be considered as the fourth pillar of cancer therapy. The clinical success of immunotherapy greatly changed the field of oncology by highlighting the importance of the immune system in cancer control and elimination. It has now become clear that research into, and the clinical application of, the immune response are important for effective cancer treatment. Moreover, it has become apparent that conventional cancer treatments, such as radiotherapy and chemotherapy, can modulate the cross-talk between the tumor and the immune system, and their efficacy depends, in part, on the ability to elicit antitumor immune response. The ability of radiotherapy to induce an immune response has become relevant in the immunotherapy age. Radiotherapy has been redefined as a partner for cancer immunotherapy, based on evidence indicating the potential synergistic effect of the combination of these therapeutic modalities. This review outlines the major findings reported to date on the immune response induced by radiotherapy and discusses the role of radiotherapy in combination with immunotherapy. Furthermore, we introduce research aimed at the clinical application of combination therapy and discuss its potential in clinical practice and future issues.

## Teaser

Radiotherapy is under investigation as an optimal partner of cancer immunotherapy due to its immune stimulatory effects and potential for enhancing the efficacy of immunotherapy.

## Introduction

In the age of immunotherapy, which is represented by immune checkpoint inhibitors (ICIs), the importance of the immune system for the elimination of cancer has become clear. Cancer cells acquire the ability to escape immune control through a process called ‘immunoediting’ before they develop into clinical cancer (1). Furthermore, it has been reported that a similar immune escape process contributes to the mechanism of resistance to immunotherapy (2), suggesting that clinically evident tumor is potentially refractory to immunotherapy. Therefore, if a tumor-specific immune response can be effectively generated both locally and systemically in patients with cancer, it may be possible to overcome this immunosuppressive scenario. In this context, a new role is emerging for radiotherapy in overcoming immunosuppression in the tumor microenvironment.

Radiotherapy is one of the three pillars of cancer therapy, together with surgery and chemotherapy, and has been widely used for the treatment of various types of cancer, both in a curative and palliative manner. To date, its therapeutic effect has been considered to be mainly local and to occur via the direct or indirect DNA damage of irradiated cancer cells. However, accumulating preclinical and clinical evidence now indicates that radiotherapy has systemic antitumor effects that are exerted through changes in the immune environment. In fact, the systemic immune response induced by radiotherapy is thought to be responsible for the so-called abscopal effect, which consists in the shrinkage of tumors outside the irradiation field (3,4). The abscopal effect has a long history, with its first report dating back to 1953 (5). This was a very rare phenomenon in the setting of radiotherapy alone (6). However, in the age of immunotherapy, abscopal effects have been increasingly reported in patients treated with radiotherapy due to progression on immune checkpoint blockade therapy (7). Thus, radiotherapy is under investigation as a modality that can enhance the efficacy of immunotherapy. The potential of radiotherapy used with immunotherapy to foster systemic tumor regression in advanced stage cancers with distant metastases has opened a new filed of investigation into the mechanisms of the underlying immune response induced by radiotherapy. Importantly, it should be noted that a prospective randomized clinical trial of immunotherapy alone versus the combination of radiotherapy with the same immunotherapy is required to assess accurately the abscopal effect of local radiotherapy plus immunotherapy (4). The shrinkage of tumors outside the irradiation field in a single-arm combination of radiotherapy and immunotherapy should not be interpreted as an abscopal effect, if the tumor type treated is known to be responsive to the immunotherapy used.

Recent advances in therapeutic technology enable the delivery of more accurate radiotherapy, in addition to conventional treatments using X-rays (3D conformal radiotherapy). This technological advancement allows the reduction of the irradiation dose to the normal tissue around the target (i.e. the primary lesion or metastatic lesion), reducing unwanted side effects. In the current radiotherapy setting, it is possible to select the treatment modality, including intensity-modulated radiotherapy (IMRT), stereotactic body radiotherapy (SBRT) and particle beam therapy (heavy ion radiotherapy, proton therapy), on a case-by-case basis considering the target site, disease stage and patient background. This advancement in treatment technology has allowed the safe administration of high doses of radiation, thus improving local control rates while enhancing the importance of controlling tumors outside the irradiation field for the long-term survival of patients.

The application of ICIs, which are currently attracting the greatest amount of attention in oncology field, achieved high efficacy (such as complete response) in a restricted number of patients (8,9). Therefore, the combination of radiotherapy and immunotherapy may be an effective complementary strategy, because radiotherapy can assist immunotherapy by enhancing immune activation both systemically and locally, whereas immunotherapy can enhance the immune response induced by local radiotherapy. In this review, we discuss the data supporting this combinatorial therapeutic strategy and the role of radiotherapy in the age of the rapid expansion of cancer immunotherapy.

## Immune responses induced by the DNA damage of irradiation

Conventionally, the therapeutic mechanism underlying the effects of radiotherapy has been considered to be the induction of direct or indirect DNA damage, which leads to cancer cell death. However, it is now recognized that the induction of an antitumor immune response by radiotherapy also contributes to its antitumor effects (10). Furthermore, evidence accumulated over recent years has revealed that this radiation-induced DNA damage itself triggers the recognition of cancer cells by the immune system.

The cyclic GMP–AMP (cGAMP) synthase (cGAS)—stimulator of interferon genes (STING) pathway plays a crucial role in the DNA damage-induced immune response. Cytoplasmic double-strand DNA of nuclear and mitochondrial origin increases as the result of radiation-induced DNA damage and binds to cGAS, followed by the catalysis of the synthesis of cGAMP, which is a secondary messenger that binds to and activates the adaptor protein STING (11–14). The activated cGAS/STING pathway eventually leads to the generation of the type-I interferon (IFN-I) mRNA via IRF3/NF-κB-dependent transcriptional activation and the induction of IFN-stimulated genes (15–17). The IFN-I generated by the cGAS/STING pathway induces dendritic cell migration to the tumor and cross-priming of T cells, which are required for the antitumor effect of radiotherapy (18). Activation of the cGAS/STING pathway is regulated by TREX1, which is an abundant intracellular 3′ → 5′ exonuclease that degrades cytoplasmic single- or double-stranded DNA (19). TREX1 regulates the immune response by suppressing IFN-I production; its deficiency leads to autoimmune diseases in humans and mice (20). Importantly, the balance between cGAS/STING pathway activation and DNA damage-initiated TREX1 induction after irradiation is affected by the radiation dose. Preclinical data suggest that hypofractionated radiation given as a few doses in the range of 8–12 Gy per fraction activates the cGAS/STING pathway more effectively than higher single doses of 20 Gy or more, at least in part due to the upregulation of TREX1 (13). TREX1-mediated degradation of cytosolic DNA hinders the activation of the cGAS/STING pathway in the cancer cells themselves as well as in dendritic cells that uptake extracellular vesicles produced by the irradiated cancer cells (21). The role of TREX1 in response to radiotherapy used in combination with immunotherapy in the clinic remains to be elucidated. However, in a recent phase I study of multi-site radiotherapy and pembrolizumab in which serial biopsies of the irradiated tumors were evaluated, increased TREX1 post-radiation was associated with poor tumor response (22). The mechanisms regulating the levels of cytosolic DNA and TREX1 activity in response to radiation, including DNA damage repair factors or cell cycle checkpoints, still need to be elucidated. Nevertheless, the key role of TREX1 in regulation of type-I interferon activation in autoimmunity suggests that it could be a therapeutic target to increase the radiation-induced induction of antitumor immune responses (23). Conversely, IFN-I, as well as type-II interferon, upregulates the immune checkpoint protein, programmed death ligand 1 (PD-L1) (24). Taken together, these findings suggest that IFN induction via the cGAS/STING pathway, which is triggered by radiation-induced DNA damage, may ultimately have dual immune activating and immunosuppressive effects.

The DNA damage caused by radiotherapy also enhances the expression of major histocompatibility complex (MHC) class I molecules in cancer cells. Reits et al. demonstrated that irradiation induced a dose-dependent increase in the levels of intracellular peptides derived from existing proteins, as well as newly increased protein synthesis via mTOR activation (25). Interestingly, in their analysis, the peptides that were uniquely expressed in irradiated cells and presented by MHC class I molecules were derived from proteins involved in DNA repair. These results suggest that the mechanism underlying the radiation-induced expression of MHC class I molecules may be involved in the promotion of DNA repair, as well as the enhancement of existing protein degradation and new protein synthesis.

The induction of the expression of PD-L1 by radiotherapy has been reported in a few studies and mostly attributed to inflammatory cytokines (26,27). We elucidated the mechanism by which the DNA damage signal itself regulates PD-L1 expression. We found that DNA double-strand breaks (DSBs) induced PD-L1 expression in a transcription-dependent manner via DNA damage signaling molecules, including ataxia telangiectasia mutated and ataxia telangiectasia and Rad3-related (ATR), Chk1 and the downstream STAT-IRF1 pathway (28). Furthermore, the depletion of DNA repair proteins, including Ku80 and breast invasive carcinoma 2 (BRCA2), enhanced PD-L1 upregulation induced by DSBs. These results suggest that radiotherapy or DNA damaging chemotherapy may highly induce PD-L1 expression in patients with mutations in these proteins; therefore, PD-L1 inhibition as a consolidation therapy after radiotherapy or chemotherapy may be effective in these cases. In support of our results, the analysis of a mouse model demonstrated that ATR inhibition suppresses PD-L1 expression in cancer cells and enhances the cell-killing effect of T cells (29). Furthermore, a significant delay in tumor growth was observed in combination of ATR inhibitor with radiotherapy (30). Whereas PD-L1 expressed on the surface of cancer cells inhibits activation of T cells by interacting with PD-1, a role that forms the basis for its therapeutic targeting by antibodies (31), a recent report highlights another role of intracellular PD-L1 in stabilizing the mRNA of DNA damage/repair proteins (e.g. NBS1 and BRCA1) (32). This intriguing finding suggests that intracellular PD-L1 may contribute to the therapeutic resistance of cells that survive radiotherapy. Therefore, strategies to inhibit intracellular PD-L1 may enhance the effect of radiotherapy and chemotherapy by inhibiting DNA repair.

In summary, DNA damage, which was previously thought to be the central mechanism underlying the cell-killing effect of radiotherapy, not only induces direct cell death but also modulates the cross-talk between the immune system and the tumor. For its combination with immunotherapy, further research is needed to establish better combination strategies through the elucidation of the radiotherapy-induced DNA damage/repair signals that enhance immune activation. This may also lead to the discovery of new drugs for targets involved in DNA damage/repair, which will enable more effective combination therapies.

## Immune responses induced by irradiation in the tumor microenvironment

One of the important effects of radiotherapy on the immune environment is a form of cell death that is called immunogenic cell death (ICD) (33). The release of damage-associated molecular patterns that promote the migration and activation of dendritic cells and antigen presentation to CD8-positive T cells is an important feature of ICD. ICD includes the expression of calreticulin on the cell surface, the release of high-mobility group protein box 1 (HMGB1) and adenosine-5′-triphosphate (ATP) (34). Calreticulin is a chaperone that is present on the surface of the endoplasmic reticulum; moreover, its expression on the cell membrane promotes the phagocytosis of irradiated cells by dendritic cells (35). HMGB1 is a non-histone chromosomal protein that, when released extracellularly, acts as an agonist of the toll-like receptor 4 on the surface of dendritic cells, thus activating them to induce an antigen-specific T-cell response (36,37). ATP activates dendritic cells by binding to their P2X7 receptors and results in IFNγ-producing CD8-positive T cells priming (38). In addition, the tumor-associated antigens released during ICD are taken up by dendritic cells that cross-present them to CD8+ T cells (39,40). Tumor DNA carried by exosomes produced by irradiated cancer cells contributes to dendritic cell activation by stimulating IFN-I production via the cGAS/STING pathway (21). Tumor-derived DNA is transferred to dendritic cells that phagocytize the tumor cell-derived exosomes, promoting the recognition of irradiated tumors by the immune system (41).

The results of clinical trials of radiotherapy combined with ipilimumab (anti-CTLA-4 antibody) in patients with metastatic non-small cell lung cancer (NSCLC), where ipilimumab has little single-agent activity, identified some of the factors that may determine the success of this combination therapy. Formenti et al. (42) demonstrated that, at the time of radiotherapy completion, IFN-β in the peripheral blood was significantly elevated in patients who exhibited the abscopal effect after radiotherapy to one of the metastases combined with ipilimumab therapy. In contrast, there was no significant increase in IFN-β in patients who showed progressive disease. These data suggest that the ability of radiotherapy to induce IFN-β may contribute to the success or failure of the combination therapy. A detailed analysis revealed the expansion of two CD8-positive T-cell clones that specifically recognized a mutated neoantigen encoded by the *KPNA2* gene, which is upregulated by radiation, in a patient with complete response. Together, these findings suggest that induction of IFN-I and exposure of immunogenic mutations may be important mechanisms that contribute to the success of combinations of radiotherapy with immunotherapy.

Although naïve T cells are known to be very radiosensitive, raising a concern that radiotherapy may deplete T cell infiltrating the tumor at the time of treatment, a recent analysis using a long-term image acquisition method in mice showed that most effector T cells that were present in tumors prior to radiotherapy survived radiation administered at clinical doses (43). Furthermore, T cells that survived irradiation retained their activity and their ability to produce IFNγ and kill cancer cells. Several clinical studies analyzed the infiltration of CD8-positive T lymphocytes into tumor tissues using specimens from patients. In most of these studies, specimens were collected about 1 month after the preoperative (chemo)radiotherapy. For example, infiltrating CD8-positive T cells were increased in the surgical specimens after preoperative chemoradiotherapy for NSCLC (44), esophageal cancer (45) and colorectal cancer (46–49). In contrast, the opposite result has also been reported. CD8-positive T-cell infiltration was decreased in oral squamous cell carcinoma (50) and cervical cancer (51) treated with chemoradiotherapy. Because the tumor specimens that were collected after surgery were commonly treated by radiotherapy with concurrent chemotherapy, which affect systemic lymphocytes, and approximately 1 month had elapsed since the completion of preoperative (chemo) radiotherapy, these results may not directly reflect the impact of radiotherapy on the infiltration of CD8-positive T cells. Interestingly, a recent study analyzed specimens that were collected during chemoradiotherapy. Dorta-Estremera et al. analyzed the proportion of CD8-positive T cells among tumor-infiltrating lymphocytes in cervical cancer treated with chemoradiotherapy. They reported that the number of these cells was decreased, whereas the proportion of CD69-positive activated T cells among CD8-positive T cells was increased over time (52), suggesting that chemoradiotherapy has the potential to enhance tumor infiltration by activated T cells, at least during treatment. Thus, although T cells in the irradiated field were reported to be able to survive, because chemotherapy or standard fractionated radiotherapy with a large field conventionally induces systemic lymphopenia (53), and T-cell infiltration changes over time, further clinical studies are required to clarify the optimal timing of the combination of radiotherapy, immunotherapy and chemotherapy to maximize the effect of this approach.

As described above, radiotherapy has the ability to convert the irradiated tumor into an ‘*in situ* vaccine’. Immunotherapy exerts its effect by promoting the immune response that is inherent to the host. The use of the patients’ own tumor as a source of tumor-specific antigens in radiotherapy can diversify the tumor-specific T-cell response (54,55). This activation of the immune response by radiotherapy creates an environment in which the immunotherapy can function more effectively.

## Clinical application of the combined therapy

### Published evidence

**Figure 1.**
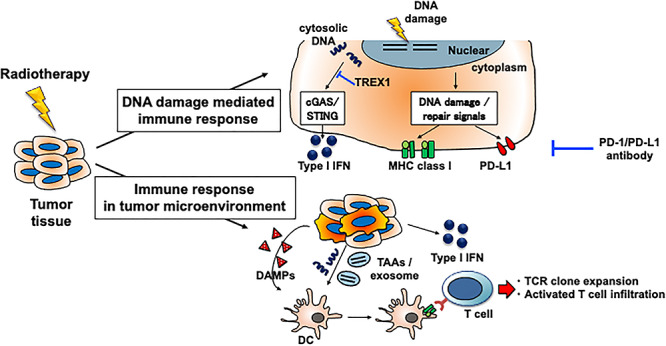
Immune responses induced by radiotherapy include those caused by DNA damage and those that occur in the tumor microenvironment. The immune response to radiotherapy creates an environment in which immune checkpoint inhibitor can more effectively eliminate tumors. Immune response to DNA damage includes programmed death ligand 1 (PD-L1) upregulation, which contributes to immunosuppression, but can be a target of the PD-1/PD-L1 blockade. DAMP, damage-associated molecular pattern; DC, dendritic cell; IFN, interferon; MHC, major histocompatibility complex; TAA, tumor-associated antigen.

Based on the accumulation of the abovementioned preclinical data (Fig. 1), the combination of radiotherapy with immunotherapy has expanded rapidly since the advent of ICIs. In the clinical studies performed to date, this combination therapy was broadly divided into strategies aimed at the cure of localized tumors and at eliciting systemic effects in metastatic cancers. An excellent example of the former is the so-called PACIFIC trial, which is a phase III clinical trial of durvalumab (an anti-PD-L1 antibody) as consolidation therapy after radical chemoradiotherapy for stage III NSCLC. This clinical study showed a significant prolongation of overall survival (OS) and progression-free survival (PFS) without a significant increase in adverse events in the treatment of stage III NSCLC in the arm given durvalumab (56,57). The PACIFIC trial does not allow the evaluation of the contribution of radiotherapy since all patients received standard-of-care chemoradiation. It is intriguing to consider if the elucidation of the optimal radiotherapy dose and fractionation to be used in this setting could further improve patients’ outcome.

In metastatic cancer, a randomized phase III trial was performed in patients with metastatic castration-resistant prostate cancer. Patients with at least one bone metastasis that had progressed after docetaxel treatment were randomly divided into two groups, i.e. bone-directed palliative radiotherapy followed by either ipilimumab or placebo therapy. A significantly longer median PFS and higher OS rate at 2–5 years were observed in the ipilimumab group (58,59). It should be noted that the results of this trial were calculated by an exploratory analysis using piecewise hazard model because the OS curves were crossed at 7–8 months and the proportional hazard ratio assumption was not met, but it is pointed out to be inappropriate for this study (60). Therefore, an alternative method of re-evaluation by mean survival time (MST) was suggested, which showed that the MST up to 52 months was significantly longer in the ipilimumab group compared with the placebo group (60).

**Table 1:** Active (i.e. ‘Recruiting’ and ‘Active, not recruiting’ in ClinicalTrials.gov) phase III clinical trials of immune checkpoint inhibitors combined with (chemo)radiotherapy

Primary lesion	Study title	Radiotherapy	Immune checkpoint inhibitor	Design	NCT number
**Anti-PD-1/PD-L1 antibody**					
Head and neck	Study of Pembrolizumab Given Prior to Surgery and in Combination With Radiotherapy Given Post-surgery for Advanced Head and Neck Squamous Cell Carcinoma (MK-3475-689)	EBRT (60 Gy/30 fr. or 66 Gy/33 fr. or 70 Gy/35 fr.)	Pembrolizumab	Experimental: Pembrolizumab > surgery > Pembrolizumab + CRT Comparator: Surgery > CRT	NCT03765918
	Study of Pembrolizumab (MK-3475) or Placebo With Chemoradiation in Participants With Locally Advanced Head and Neck Squamous Cell Carcinoma (MK-3475-412/KEYNOTE-412)	EBRT (70 Gy/35 fr.)	Pembrolizumab	Experimental: Pembrolizumab > CRT + Pembrolizumab > Pembrolizumab Comparator: Placebo > CRT + Placebo > Placebo	NCT03040999
	Testing Immunotherapy Versus Observation in Patients With HPV Throat Cancer	IMRT (70 Gy/35 fr.)	Nivolumab	Experimental (Arm A): CRT > Nivolumab Comparator (Arm B): CRT Experimental (Arm C): Nivolumab	NCT03811015
	Sintilimab (PD-1 Antibody) and Chemoradiotherapy in Locoregionally advanced Nasopharyngeal Carcinoma	IMRT (70 Gy in 6–7 weeks)	Sintilimab	Experimental: chemotherapy + Sintilimab > CRT + Sintilimab Comparator: Chemotherapy > CRT	NCT03700476
	Programmed Death-1 (PD-1) Antibody Combined With IMRT in Recurrent Nasopharyngeal Carcinoma Patients	IMRT (60–66 Gy/30–33 fr.)	Toripalimab	Experimental: RT + toripalimab Comparator: RT alone	NCT03907826
	Toripalimab Plus Concurrent Chemo-radiotherapy for Unresectable Locally Recurrent Nasopharyngeal Carcinoma	IMRT: (60–64 Gy/27 fr.)	Toripalimab	Experimental: CRT + toripalimab Comparator: CRT	NCT04453813
	Radiation Therapy With Durvalumab or Cetuximab in Treating Patients With Locoregionally Advanced Head and Neck Cancer Who Cannot Take Cisplatin	IMRT	Durvalumab	Experimental: IMRT + Durvalumab Comparator: IMRT + cetuximab	NCT03258554
	Concurrent and Adjuvant PD1 Treatment Combined With Chemo-radiotherapy for High-risk Nasopharyngeal Carcinoma	IMRT	Camrelizumab	Experimental: Camrelizumab + CRT Comparator: CRT	NCT04453826
Lung	PD-1 Inhibitors and Chemotherapy With Concurrent Irradiation at Varied Tumour Sites in Advanced Non-small Cell Lung Cancer	EBRT (at least 18 Gy/3 fr.)	Pembrolizumab	Experimental: Pembrolizumab + CRT Comparator: Pembrolizumab + chemotherapy	NCT03774732
	Efficacy and Safety Study of Stereotactic Body Radiotherapy (SBRT) With or Without Pembrolizumab (MK-3475) in Adults With Medically Inoperable Stage I or IIA Non-Small Cell Lung Cancer (NSCLC) (MK-3475-867/KEYNOTE-867)	SBRT (45–54 Gy/3–5 fr.)	Pembrolizumab	Experimental: SBRT + Pembrolizumab Comparator: SBRT + placebo	NCT03924869
	Study of Pembrolizumab With Concurrent Chemoradiation Therapy Followed by Pembrolizumab With or Without Olaparib in Stage III Non-Small Cell Lung Cancer (NSCLC) (MK-7339-012/KEYLYNK-012)	EBRT (60 Gy/30 fr.)	Pembrolizumab	Experimental: Pembrolizumab + concurrent CRT > Pembrolizumab + placebo Experimental: Pembrolizumab + concurrent CRT > Pembrolizumab + olaparib Comparator: CRT > durvalumab	NCT04380636
	Immunotherapy With or Without SBRT in Patients With Stage IV Non-small Cell Lung Cancer	SBRT	Pembrolizumab	Experimental: SBRT + Pembrolizumab Comparator: Pembrolizumab alone	NCT03867175
	Durvalumab vs. Placebo Following Stereotactic Body Radiation Therapy in Early Stage Unresected Non-small Cell Lung Cancer Patients (PACIFIC-4)	SBRT	Durvalumab	Experimental: SBRT > Durvalumab Comparator: SBRT > Placebo	NCT03833154
Esophagus	Study of Pembrolizumab (MK-3475) Versus Placebo in Participants With Esophageal Carcinoma Who Are Receiving Chemotherapy and Radiation Therapy (MK-3475-975/KEYNOTE-975)	EBRT (50 Gy/25 fr. or 60 Gy/30 fr.)	Pembrolizumab	Experimental: Pembrolizumab > Pembrolizumab + CRT Comparator: Placebo > Placebo + CRT	NCT04210115
	Study of Camrelizumab (SHR-1210) in Combination With Concurrent Chemoradiotherapy in Locally Advanced Esophageal Cancer	NS	Camrelizumab	Experimental: Camrelizumab + Paclitaxel + Cisplatin + radiotherapy Comparator: Placebo + Paclitaxel + Cisplatin + radiotherapy	NCT04426955
Liver	Combination of Sintilimab and Stereotactic Body Radiotherapy in Hepatocellular Carcinoma (ISBRT01)	SBRT (30–54 Gy/3–6 fr.)	Sintilimab	Experimental: SBRT > Sintilimab Comparator: SBRT	NCT04167293
Uterine cervix	Study of Durvalumab With Chemoradiotherapy for Women With Locally Advanced Cervical Cancer (CALLA)	EBRT + brachytherapy	Durvalumab	Experimental: durvalumab + CRT > durvalumab Comparator: placebo + CRT	NCT03830866
	Study of Chemoradiotherapy With or Without Pembrolizumab (MK-3475) For The Treatment of Locally Advanced Cervical Cancer (MK-3475-A18/KEYNOTE-A18/ENGOT-cx11)	EBRT (45–50 Gy/23–28 fr.) + brachytherapy (25–30 Gy/4–6 fr.)	Pembrolizumab	Experimental: Pembrolizumab + CRT Experimental: Placebo + CRT	NCT04221945
Intestine	PD1 Antibody Sintilimab ± Chemoradiotherapy for Locally Advanced Rectal Cancer	EBRT (50 Gy/25 fr.)	Sintilimab	Experimental: Sintilimab > surgery or watch and wait > Sintilimab ± chemotherapy Experimental cohort B (arm-1): Sintilimab + CRT > surgery or watch and wait > chemotherapy Comparator cohort B (arm-2): CRT > surgery or watch and wait > chemotherapy	NCT04304209
Skin	Pembrolizumab Versus Placebo Following Surgery and Radiation in Participants With Locally Advanced Cutaneous Squamous Cell Carcinoma (MK-3475-630/KEYNOTE-630)	NS	Pembrolizumab	Experimental: surgery > radiotherapy > Pembrolizumab Comparator: surgery > radiotherapy	NCT03833167
Lymphoma	A Multicenter, Phase 3, Randomized Trial of Sequencial Chemoradiotherapy With or Without Toripalimab (PD-1 Antibody) in Newly Diagnosed Early-Stage Extranodal Natural Killer/T Cell Lymphoma, Nasal Type (ENKTL)	IMRT (54–56 Gy in 25–26 weeks)	Toripalimab	Experimental: toripalimab + chemotherapy > toripalimab + radiotherapy > toripalimab Comparator: chemotherapy > radiotherapy	NCT04365036
**Anti-PD-1/PD-L1 antibody + anti-CTLA-4 antibody**					
Brain	Testing the Use of the Immunotherapy Drugs Ipilimumab and Nivolumab Plus Radiation Therapy Compared to the Usual Treatment (Temozolomide and Radiation Therapy) for Newly Diagnosed MGMT Unmethylated Glioblastoma	NS	Nivolumab and Ipilimumab	Experimental: RT + Nivolumab + Ipilimumab Comparator: RT + Temozolomide > Temozolomide	NCT04396860
Head and neck	Study of Nivolumab Alone or in Combination With Ipilimumab as Immunotherapy vs. Standard Follow-up in Surgical Resectable HNSCC After Adjuvant Therapy	EBRT (56–66 Gy)	Nivolumab and Ipilimumab	Experimental: Nivolumab > surgery > RT or CRT > Nivolumab (arm Ia) or Nivolumab + Ipilimumab (arm Ib) Comparator: Surgery > RT or CRT	NCT03700905
Lung	Phase III Trial of (Local Consolidation Therapy; LCT) After Nivolumab and Ipilimumab	NS	Nivolumab and Ipilimumab	Experimental: Arm A (ipilimumab, nivolumab): (INDUCTION) Nivolumab + Ipilimumab > Nivolumab + Ipilimumab Experimental: Arm B (ipilimumab, nivolumab, LCT): (INDUCTION) Nivolumab + Ipilimumab > LCT (surgery and/or RT) > Nivolumab + Ipilimumab	NCT03391869
Esophagus	Nivolumab and Ipilimumab in Treating Patients With Esophageal and Gastroesophageal Junction Adenocarcinoma Undergoing Surgery	NS	Nivolumab and Ipilimumab	Experimental: Arm A: CRT Experimental: Arm B: CRT + Nivolumab Experimental: Arm C: Nivolumab Experimental: Arm D: Nivolumab + Ipilimumab	NCT03604991

EBRT, externalbeam radiotherapy; fr., fractions; CRT, chemoradiotherapy; IMRT, intensity-modulated radiotherapy; RT, radiotherapy; SBRT, stereotactic body radiotherapy; LCT, local consolidation therapy.

The results of aforementioned phase II clinical trial for patients with metastatic NSCLC showed that hypofractionated radiotherapy (6 Gy × 5 or 9 Gy × 3) to a single metastasis with initiation of ipilimumab on the first day of radiation resulted in intent-to-treat overall response rate (ORR) of non-irradiated metastases in 18% of patients, and clinical benefit in 31% of patients, without an increase in side effects due to the combination (42). Another phase II randomized trial in patients with metastatic NSCLC compared SBRT to a metastasis before the initiation of pembrolizumab (an anti-PD-1 antibody) with pembrolizumab monotherapy. The results revealed that OS, PFS and ORR of non-irradiated lesion showed improved trends in the SBRT combination group without an increase in treatment-related adverse events (61). A pooled analysis of this study and another phase I/II study of SBRT in combination with pembrolizumab demonstrates the significant superiority of the SBRT combination group in non-irradiated tumor response and control rates, OS and PFS (62). Furthermore, in a prospective study of patients with metastatic melanoma treated with radiotherapy to one or two disease sites with concurrent ipilimumab, half of all patients had a clinical benefit during the observation period, including durable CR in 3 of 22 patients (63). In another phase I trial, hypofractionated radiotherapy against a metastasis combined with pembrolizumab resulted in PR in 2 of 12 patients with NSCLC or melanoma who were previously treated with anti-PD-1 antibodies and CR in 1 of 12 patients with other tumors not previously treated with anti-PD-1 antibodies. Furthermore, importantly, there were no grade 3 or higher adverse events (64). The results of these clinical trials suggest the potential of combined therapy for both locally advanced and systemically metastatic cancer.

Several retrospective analyses also support the potential benefit of ICIs combined with radiotherapy. For example, the secondary analysis of a phase I clinical trial of pembrolizumab monotherapy for patients with locally advanced or metastatic NSCLC, the KEYNOTE-001 trial, revealed that patients who previously received any radiotherapy had a significantly longer OS and PFS compared with patients who did not receive radiotherapy (65). Similarly, in another retrospective analysis of nivolumab (an anti-PD-1 antibody) for patients with advanced NSCLC, a group of patients who had a history of radiotherapy, regardless of its radical or palliative nature, had a significantly better OS and PFS than did those without a history of radiotherapy (66). Significantly, the incidence of treatment-related pulmonary toxicity was greater in the radiation-treated group, but the incidence of grade 3 or higher pneumonitis was not significantly different between the groups in these analyses. Moreover, among patients with metastatic lung tumors treated with anti-PD-1/PD-L1 antibodies, a trend toward longer OS was reported in patients with prior thoracic radiotherapy than in those without radiotherapy, despite including fewer targetable mutations (67). Another retrospective analysis reported that palliative radiotherapy for advanced metastatic melanoma patients with progression after ipilimumab treatment resulted in an abscopal effect in 11 of 21 patients and significantly longer OS in those who showed the abscopal effect than in those who did not (68). Overall, these reports have stimulated a several clinical trials of radiotherapy combined with immunotherapy that are currently under way.

### Ongoing clinical trials

A search of ClinicalTrials.gov for phase III clinical trials aimed at investigating the efficacy of the combination of radiotherapy and ICI therapy identified 25 combined clinical trials (Table 1). Most of these trials included radiotherapy in both arms, examining the additional effect of ICI on radiotherapy. The breakdown of target organs in these clinical studies was as follows: nine trials in the head and neck region, six trials in the lung, three trials in the esophagus, two trials in the cervix and one trial each in the brain, liver, lower gastrointestinal tract, skin and lymphoma. Many clinical trials seem to be under way that target sites, such as head and neck cancer and lung cancer, which are treated by high-precision radiotherapy modalities, such as IMRT. In fact, the modality of radiotherapy in these trials was as follows: seven trials used IMRT and four trials used SBRT, which means that the so-called high-precision radiotherapy is often used in combination trials. Interestingly, with regard to the timing of the combination, most of these ongoing trials use concurrent combinations of radiotherapy with ICIs, in contrast to the PACIFIC trial, which is the sequential combination of chemoradiotherapy with conventional fractionation and anti-PD-L1 antibody therapy. The results of these studies will clarify the effectiveness of the combination strategy, which has been supported by many preclinical studies.

## Perspectives

High-precision radiotherapy has made it possible to increase the dose size of radiotherapy relatively safely, and the role of radiotherapy as an option for cancer treatment is expanding at both early and advanced stages of the disease. On considering the combination with immunotherapy, which is aimed at the local and systemic disease control, a radiotherapy protocol that maximizes the immune response must be established. Specifically, it will be useful to elucidate how DNA damage signaling affects the immune response after radiotherapy. Furthermore, the elucidation of the optimal radiotherapy method premised on the combined therapy, such as the irradiation dose, the number of fractions and the timing of the combined use of ICIs, is necessary. The survival term was significantly longer in mice that started the anti-PD-L1 antibody during fractional radiotherapy compared with those that received sequential administration after the completion of radiotherapy (26). In another mouse study, the timing of 20 Gy × 1 fraction and anti-CTLA4 antibodies reported that the best tumor control and survival advantage was observed in the group that started the anti-CTLA4 antibodies before radiotherapy, compared with the group that started it after radiotherapy (69). In clinical practice, a retrospective analysis of the timing of palliative radiotherapy and ipilimumab for metastatic melanoma reported that the median OS was 9 months in patients who received radiotherapy during induction of ipilimumab and 39 months in patients who received radiotherapy while continuing ipilimumab (70). These results suggest that ICIs should be initiated prior to or concurrently with radiotherapy, rather than after radiotherapy, although accurate evaluation requires comparison by prospective clinical trials due to differences in dosages and ICI used in these studies. Regarding the irradiation field, when it includes the draining lymph nodes, it can suppress the radiotherapy-induced immune response, as shown in preclinical studies (71). Therefore, the radiotherapy method used in the combination treatment with immunotherapy may differ from the conventional radiotherapeutic treatment strategies. Taken together, these results imply that ICI may be preferable prior to or concurrent with radiotherapy in a single 10 Gy fractionated dose that avoids draining lymph nodes. However, despite a wealth of preclinical evidence, clinical evidence that radiotherapy enhances immunotherapy responses is limited, calling for clinical studies that include an in-depth immunomonitoring to elucidate the mechanisms of success or failure in patients.

## Conclusion

The role of radiotherapy in the age of ICIs and precision medicine is evolving, and in the future, radiotherapy may be used not only as a local treatment but also as a systemic one in combination with immunotherapy. Whereas the results of recent clinical studies are promising, evidence that radiotherapy can reliably improve responses to immunotherapy is still lacking. Thus, more studies are needed to understand the immunogenic effects of radiation in preclinical models and in patients.
